# Genome Mining of *Pseudarthrobacter* sp. So.54, a Rhizospheric Bacteria from *Colobanthus quitensis* Antarctic Plant

**DOI:** 10.3390/biom15040534

**Published:** 2025-04-05

**Authors:** Dayaimi González, Pablo Bruna, María J. Contreras, Karla Leal, Catherine V. Urrutia, Kattia Núñez-Montero, Leticia Barrientos

**Affiliations:** 1 Programa de Doctorado en Ciencias Mención Biología Celular y Molecular Aplicada, Universidad de La Frontera, Temuco 4811230, Chilec.urrutia08@ufromail.cl (C.V.U.); 2 Centro de Excelencia en Medicina Traslacional (CEMT), Universidad de La Frontera, Avenida Alemania 0458, Temuco 4810296, Chile; 3 Núcleo Científico y Tecnológico en Biorecursos (BIOREN), Universidad de La Frontera, Avenida Francisco Salazar 01145, Temuco 4811230, Chile; 4 Facultad de Ciencias de la Salud, Instituto de Ciencias Aplicadas, Universidad Autónoma de Chile, Avenida Alemania 1090, Temuco 4800000, Chile; 5 Facultad de Ingeniería, Instituto de Ciencias Aplicadas, Universidad Autónoma de Chile, Avenida Alemania 1090, Temuco 4800000, Chile

**Keywords:** extreme environments, microorganisms, adaptation, whole genome, bioactive compounds

## Abstract

Antarctic microorganisms have genomic characteristics and biological functions to ensure survival in complex habitats, potentially representing bioactive compounds of biotechnological interest. *Pseudarthrobacter* sp. So.54 is an Antarctic bacteria strain isolated from the rhizospheric soil of *Colobanthus quitensis*. Our work aimed to study its genomic characteristics and metabolic potential, linked to environmental adaptation and the production of secondary metabolites with possible biotechnological applications. Whole-genome sequencing, assembly, phylogenetic analysis, functional annotation, and genomic islands prediction were performed to determine the taxonomic affiliation and differential characteristics of the strain So.54. Additionally, Biosynthetic Gene Clusters (BGCs) responsible for secondary metabolites production were identified. The assembled genome of strain So.54 has 3,871,805 bp with 66.0% G + C content. Phylogenetic analysis confirmed that strain So.54 belongs to the *Pseudarthrobacter* genus; nevertheless, its nucleotide and amino acid identity values were below the species threshold. The main metabolic pathways and 64 genomic islands associated with stress defense and environmental adaptation, such as heavy metal resistance genes, were identified. AntiSMASH analysis predicted six BGCs with low or no similarity to known clusters, suggesting potential as novel natural products. These findings indicate that strain So.54 could be a novel *Pseudarthrobacter* species with significant environmental adaptation and biotechnological potential.

## 1. Introduction

*Pseudarthrobacter*, a genus of the family *Micrococcaceae*, which belongs to the phylum *Actinobacteroidota*, comprises aerobic, motile, and Gram-positive bacteria [1]. This genus is classified outside the *Arthrobacter* genus due to differences in phylogenetic position and chemotaxonomic traits, including the polar lipid profile, quinone system, and peptidoglycan type, between the two groups [2,3]. However, both genera have similar genomes that contain features enabling survival in adverse environmental conditions, such as the Antarctic. This continent is considered the coldest, driest, and windiest environment on earth [4]. These extreme conditions have favored the evolution and adaptation of microorganisms in the Antarctic.

Previous studies have described that *Pseudarthrobacter* sp. has functional genes that resist desiccation, low temperatures and nutritional levels, and high irradiation conditions [5,6]. Some of these genes are related to membrane transport, stress response, resistance to antibiotics and toxic compounds, the metabolism of carbohydrates and aromatic compounds, and heavy metal tolerance [7,8]. Polysaccharides, such as glycogen, trehalose, and maltodextrin, are major carbohydrates present in cold-adapted microorganisms, and bacteria, with their complete metabolic pathways, indicate the ability to conserve energy and its utilization [9]. Another survival strategy appears to be to possess a flexible cellular membrane resulting from a higher proportion of unsaturated fatty acids and a high production of catalases and superoxide dismutases that have considerable capacity to decrease higher reactive oxygen species (ROS) assembly [10].

*Actinobacteroidota* phylum members such as *Pseudarthrobacter* sp. could generate new metabolic pathways under stress conditions, allowing the synthesis of new bioactive products with unique properties and structures associated with the presence of biosynthetic gene clusters (BGCs). *Actinobacteroidota* is the most productive group of bacteria in drug discovery. It can possess dozens of BGCs to produce secondary metabolites, of which only about 10% have been described [11]. Although the genus *Pseudarthrobacter* has been widely studied, particularly regarding its efficiency in secondary metabolite production, there is still a need to explore new strains, especially those from extreme environments. While several bioactive compounds with biotechnological applications, such as antibiotics, antifungals, anti-inflammatory, antiparasitic, antitumor, antiviral, insecticidal, growth-promoting metabolites, and enzyme inhibitors, have been reported [12], further investigation is required to fully understand the metabolic capabilities of newly discovered strains and the potential biotechnological applications of the products they can produce. In this context, in the present work, we isolated a *Pseudarthrobacter* strain from Antarctic rhizospheric soil and studied its genomic characteristics associated with environmental adaptation and metabolic potential.

## 2. Materials and Methods

### 2.1. Bacterial Isolation and Cultivation

Rhizosphere soil samples were collected in triplicate between February and May 2022 from *Colobanthus quitensis* plants on King George Island, Antarctica. Three quadrants (5 × 5 m each) were established at each sampling site, representing three rhizosphere cores. Within each quadrant, three plants and their rhizosphere soil were randomly selected and combined to form a composite rhizosphere soil sample. During sampling, the rhizosphere soil of each plant was carefully extracted by excavating the root zone to a depth of 0–20 cm using a sterilized spade. Soil located approximately 2 mm from the roots was collected and placed into sterile polyethylene bags. The samples were refrigerated at 4 °C, transported to the laboratory, and stored at −80 °C until further analysis.

To isolate bacteria from previous samples, 1 g of soil was taken and diluted in 100 mL of sterile saline solution (0.9%). The mixture was then shaken for 30 min in a vortex at room temperature. Subsequently, 50 µL of the shaken mixture was plated onto Tryptic Soy Agar (TSA) (agar 15.0 g/L, pancreatic digest of casein 15.0 g/L, sodium chloride 5.0 g/L, and papaic digest of soybean 5.0 g/L), King Agar B (agar 10.0 g/L, dipotassium hydrogen phosphate 1.5 g/L, magnesium sulfate 1.5 g/L, and mixed peptone 20.0 g/L), and R2A (yeast extract 0.5 g/L, protease peptone 0.5 g/L, casein hydrolysate 0.5 g/L, glucose 0.5 g/L, starch soluble 0.5 g/L, sodium pyruvate 0.3 g/L, di-potassium hydrogen phosphate 0.3 g/L, magnesium sulfate anhydrous 0.024 g/L, and agar-agar 15.0 g/L) culture media plates in serial dilutions ranging from 10^−2^ to 10^−10^. The cultures were incubated for 4 weeks at 15 °C. The resulting colonies were isolated and subsequently plated until purification in Luria Bertani (LB) agar medium (bacteriological agar 15.0 g/L, tryptone 10.0 g/L, sodium chloride 10.0 g/L, and yeast extract 5.0g/L). Pure cultures were preserved by freezing at −80 °C in glycerol (20% *v*/*v*) until further use. The strain used in this study was deposited in the Colección Chilena de Cultivos Tipo—CCCT (Universidad de La Frontera) under accession code So.54.

For DNA extraction, the isolated bacterial strain was inoculated on LB agar and incubated at 15 °C for seven days until suitable microbial growth was observed.

### 2.2. DNA Extraction

Genomic DNA extraction was performed using the AccuPrep **^®^** Genomic DNA Extraction Kit (K-3032G, BIONEER, Daejeon, Republic of Korea) following the manufacturer’s instructions. DNA quality was assessed by fluorescence quantification using the Qubit dsDNA HS Assay Kit (Invitrogen, Waltham, MA, USA), and a final DNA concentration greater than or equal to 50 ng/μL was selected. Integrity was verified by 1% agarose gel and purity according to A260/A280.

### 2.3. Genome Sequencing and Assembly of Pseudarthrobacter sp. So.54

The genome sequencing was performed using Oxford Nanopore Technologies (ONT) technologies. The ONT library was prepared using the Rapid Sequencing kit SQK-RBK004 and sequenced on the MinION Mk1C of the Extreme Environments Biotechnology Laboratory (Universidad de La Frontera, Chile) according to the manufacturer’s recommendations and using the MinKNOW software v.4.0.20. The base-calling of the ONT reads was performed with Guppy v3.1.5 software. The quality control of readings was evaluated using the Nanoplot v1.40.0 tool [13]. After that, the adapters were cut with Porechop v.0.2.4, and the sequences with a quality more significant than ten were filtered using Nanofilt v2.8.0 [13]. De novo assembly was performed using NECAT v.0.0.1 [14] with the default tool parameters. Finally, the quality and contamination of the genomes assembled with the Quast v5.0.2 and CheckM v1.1.3 tools were evaluated [15,16]. The genome assembly generated in this study is available in the National Center for Biotechnology Information (NCBI) under the BioProject ID: PRJNA1240362.

### 2.4. Phylogenetic Analysis

The Type Strain Genome Server (TYGS) was used to analyze the phylogeny [17]. First, the So.54 genome was compared against all type strain genomes available in the TYGS database via the MASH algorithm, a fast approximation of intergenomic relatedness [18], and the type strains with the smallest MASH distances were chosen. Second, the 16S rDNA gene sequence of the So.54 strain was extracted using RNAmmer v1.2 [19] and subsequently BLASTed using BLAST+ v2.16.0 (NCBI) with databases in v5 format [20]. All pairwise comparisons among the genomes were conducted using the Genome BLAST Distance Phylogeny (GBDP) approach and accurate intergenomic distances were inferred under the algorithm ’trimming’ and distance formula d5 [21] (https://tygs.dsmz.de, accessed on 08 June 2024). The resulting intergenomic distances were used to infer a balanced minimum evolution tree with branch support via FASTME 2.1.6.1, including SPR postprocessing [22]. Branch support was inferred from 100 pseudo-bootstrap replicates each. The tree was rooted at the midpoint and visualized with PhyD3 [23]. The type-based species clustering using a 70% digital DNA-DNA hybridization (dDDH) radius around each of the 15 type strains was performed as previously described [17], and subspecies clustering was conducted using a 79% dDDH threshold [24]. Also, the pairwise whole genome comparisons of average nucleotide identity (ANI) and average amino acid identity (AAI) were calculated according to Konstantinidis and Tiedje using the scripts available at http://enve-omics.gatech.edu/(accessed on 12 June 2024) [25].

### 2.5. Genomic Bioinformatics Analysis

A circular map of the So.54 genome was prepared using the PROKSEE server v1.1.2 [26]. The draft genome sequence was annotated using the Rapid Annotation Subsystem Technology (RAST) server, which is integrated with SEED Viewer v2.0 [27]. The RAST server annotated and classified predicted genes according to function. The genes were categorized into two groups: either in the subsystem or not in the subsystem, depending on the protein families with common function. Genes that were categorized in the subsystem were considered reliable and conservative gene predictions. Genome annotation was performed using PROKKA v1.14.6, which utilized UniProt, RefSeq, and a series of hidden Markov model profile databases, including Pfam and TIGRFAMs [28], and the amino acid sequences were submitted to the Kyoto Encyclopedia of Genes and Genomes (KEGG). The functional categories analysis and metabolic pathways were determined using BlastKOALA v3.0 in KEGG [29] and Anvi’o v7 [30]. Further, CAZyme analysis was conducted with the help of dbCAN3 server, selecting three tools that comprise HMMER: dbCAN (E-Value < 1 × 10^−15^, coverage > 0.35), DIAMOND: CAZy (E-Value < 1 × 10^−102^) and HMMER: dbCAN-sub (E-Value < 1 × 10^−15^, coverage > 0.35) [31].

Genomic island prediction was performed with IslandViewer v4 via the IslandPath-DIMOB, SIGI-HMM, and IslandPick methods [32]. The antiSMASH v6.1.1 server was employed to predict all gene clusters in the strain So.54, focusing on the biosynthesis of new metabolites with biotechnological potential. The relaxed detection parameter was used, including the prediction features “KnownClusterBlast”, “MIBiG cluster comparison”, “Cluster Pfam analysis”, “ClusterBlast”, “ActiveSiteFinder”, “Pfam-based GO term annotation”, “SubClusterBlast”, “RREFinder”, and “TIGRFam analysis” [33].

## 3. Results

### 3.1. General Genomic Features of Strain So.54

**Figure 1:**
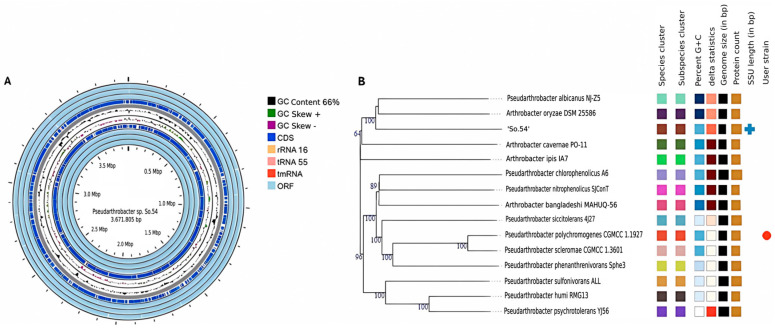
(**A**) Genome circle map of strain *Pseudarthrobacter* sp. So.54. GC content (black), GC skew curves (+/−, green/purple), coding sequences (CDSs, dark blue), rRNAs (light orange), tRNAs (pink), tmRNAs (vermilion), and open reading frames (ORFs, light blue); (**B**) Phylogenetic tree was constructed based on the whole genome using the Genome-BLAST distance phylogenetic method (GBDP) tool. According to the GBDP distance formula d5, the branch lengths were scaled.

We sequenced and assembled the complete genome of strain So.54 in one unique contig with a total length of 3,871,805 base pairs (bp) and a G + C content of 66.0%. Using Proksee, a circular map of this genome was obtained, as shown in Figure 1A. The genomic annotation revealed a predicted number of protein-coding genes (CDSs) of 4732, including hypothetical protein, and the total numbers of tRNA and rRNA were 55 and 16, respectively. The results from phylogenetic analysis using TYGS showed that strain So.54 was closely related to *Pseudarthrobacter* albicanus NJ-Z5 and *Arthrobacter oryzae* DSM 25,586 as shown in Figure 1B. These results confirmed that this strain belongs to the *Micrococcaceae* family and *Actinobacteroidota* phylum. Additionally, the strain So.54 exhibited the highest dDDH, ANI, and AAI values with *P. albicanus* NJ-Z5 (25.3% dDDH, 83% ANI, 81% AAI) and *A. oryzae* DSM 25,586 (24.6% dDDH, 83% ANI, 82% AAI) (Table S1, Figure S1). However, these values were relatively low, suggesting that the Antarctic strain So.54 might be a novel species of the genus *Pseudarthrobacter*.

**Figure 2:**
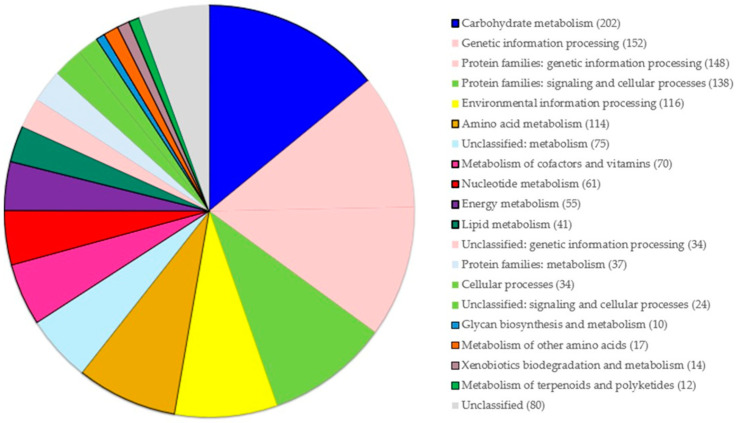
Functional categories analysis of *Pseudarthrobacter* sp. So.54. Genes involved in metabolic pathways are highlighted with black borders. The results were based on BlastKOALA in the Kyoto Encyclopedia of Genes and Genomes (KEGG).

Genomic functional analysis was performed using the KEGG database. The approximate functional distribution of strain So.54 was obtained. Among 4546 genes, 31.5% (1434) of them were annotated. Gene functions were divided into four categories by the KEGG database: metabolism (708 genes), genetic information processing (334 genes), cellular process (196 genes), and environmental information processing (116 genes), and then each category was further subdivided into subcategories (Figure 2). The main genetic content was involved in metabolic pathways of carbohydrates (202 genes), amino acids (114 genes), cofactors and vitamins (70 genes), nucleotides (61 genes), energy (55 genes), lipids (41 genes), other amino acids (17 genes), terpenoids and polyketides (12 genes), and glycan (10 genes). Furthermore, 14 genes were related to the xenobiotics biodegradation and metabolism pathway. The results confirmed that strain So.54 has strong carbohydrate metabolism, which might be related to environmental adaptation and complex carbohydrates’ degradation, transformation, and utilisation.

### 3.2. Metabolic Features and Genes Related to Environmental Adaptation

Metabolic reconstruction indicated that strain So.54 possesses several genes involved in pathways related to environmental adaptation (Table S2). The energy metabolism was associated with carbon fixation (M00165, reductive pentose phosphate cycle, and M00579, phosphate acetyltransferase-acetate kinase pathway) and the metabolism of methane (M00345, formaldehyde assimilation) and sulfur (M00176, assimilatory sulfate reduction). Two complete pathways to ATP synthesis were identified, involving cytochrome c oxidase (M00155) and F-type ATPase (M00157). Additionally, fatty acid metabolism is associated with the obtention of energy through beta-oxidation for acyl-CoA synthesis (M00086). Related to amino acid metabolism, we identified a complete GABA (gamma-aminobutyrate) shunt pathway (M00027). For the metabolism of cofactors and vitamins, a thiamine salvage pathway (M00899) and biosynthesis pathways for Pyridoxal-P (M00916), NAD (M00115), coenzyme A (CoA) (M00120), lipoic acid (M00881), and heme (M00926) were completed in the strain So.54 genome sequence. Furthermore, a complete pathway for C1-unit interconversion (M00140) was identified.

The strain So.54 only had two blocks missing in the non-mevalonate pathway for the biosynthesis of C5 isoprenoids (M00096). This strain also has the potential to degrade xenobiotics such as toluene (M00538), xylene (M00537), catechol (M00568), and phenylacetate (M00878). Thirteen genes were related to these pathway modules, although none were complete. With regards to drug resistance, this strain has the gene for beta-lactam resistance (M00627, beta-lactamase class A (EC 3.5.2.6)) and multidrug resistance associated with multidrug efflux pumps (M00639, MexCD-OprJ; M00769, MexPQ-OpmE; and M00714, QacA).

Specifically regarding carbohydrate metabolism, this strain posses the Embden–Meyerhof pathway of glycolysis (M00001), gluconeogenesis (M00003), the citrate cycle (Krebs cycle) (M00009), pentose phosphate pathway (oxidative (M00006) and nonoxidative phases (M00007)), the Leloir pathway of galactose degradation (M00632), and the semi-phosphorylative Entner-Doudoroff pathway (M00308) as an interesting metabolic strategy to degradate glucose and survive in different environmental conditions. We also identified genes related to the glyoxylate cycle (M00012), a metabolic variation of the Krebs cycle. In addition, this strain possesses metabolic pathways for the biosynthesis and degradation of polysaccharides and oligosaccharides such as glycogen (M00854, M00855) and trehalose (M00565), respectively.

#### 3.2.1. CAZyme Analysis

**Figure 3:**
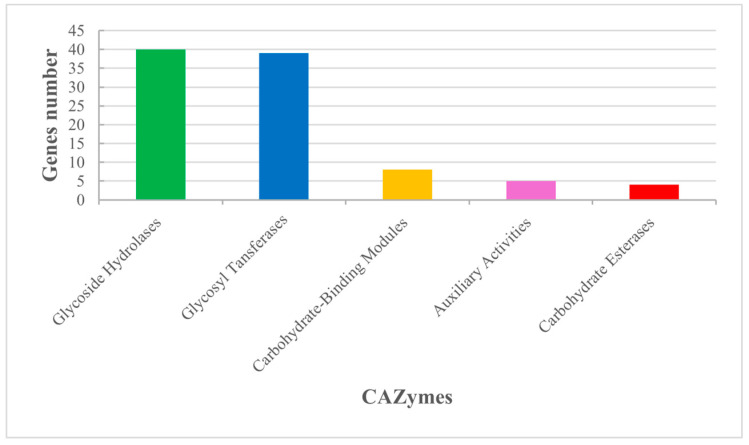
CAZy annotation classification distribution map. The horizontal axis is the CAZy category, and the vertical axis is the number of genes annotated by the corresponding category.

Based on the CAZyme annotation, the strain So.54 encodes 40 glycoside hydrolases (GH), 39 glycosyltransferases (GT), eight carbohydrate-binding modules (CBM), five auxiliary activities (AA), and four carbohydrate esterases (CE) (Figure 3). These observations indicated that glycoside hydrolases and glycosyl transferases accounted for the majority in strain So.54, providing a basis for forming, transferring, and further metabolizing monosaccharides, polysaccharides, and glycosides. We identified CAZyme members of GH and GT families involved in trehalose and glycogen metabolic pathways (Table 1).

The trehalose and glycogen metabolic pathways were evaluated using the KEGG annotation database with the Anvi’o7 platform. The strain So.54 exhibited a complete TreY/TreZ pathway (enzymes such as maltooligosyl-trehalose synthase and maltooligosyl-trehalose trehaldohydrolase) for trehalose biosynthesis, and genes corresponding to the classical path of bacterial glycogen metabolism were identified (Figure S2). This pathway includes five essential enzymes: ADP-glucose pyrophosphorylase (GlgC), glycogen synthase (GlgA), glycogen branching enzyme (GlgB), glycogen phosphorylase (GlgP), and glycogen debranching enzyme (GlgX). Additionally, the seed viewer identified all genes involved in both pathways within the RAST annotation. Therefore, we can confirm the existence of these complete pathways for trehalose and glycogen metabolism in the strain So.54.

#### 3.2.2. Stress Response Genes in the Strain So.54

Genome analysis revealed that strain *Pseudarthrobacter* sp. So.54 has multiple genes related to the response to osmotic and oxidative stress, which could be representative of its adaptation to the Antarctic environmental conditions (Table 2). Two genes (*aqpZ* and *glpF*) are related to osmoregulation in this genome, encoding the aquaporin Z and glycerol uptake facilitator protein. Moreover, the genome of strain So.54 contained eleven genes predicted to encode antioxidant enzymes such as catalase, superoxide dismutase, thioredoxins, peroxidases, organic hydroperoxide resistance proteins, and NsrR protein, which is also associated with nitrosative stress.

### 3.3. Genomic Islands Prediction

**Figure 4:**
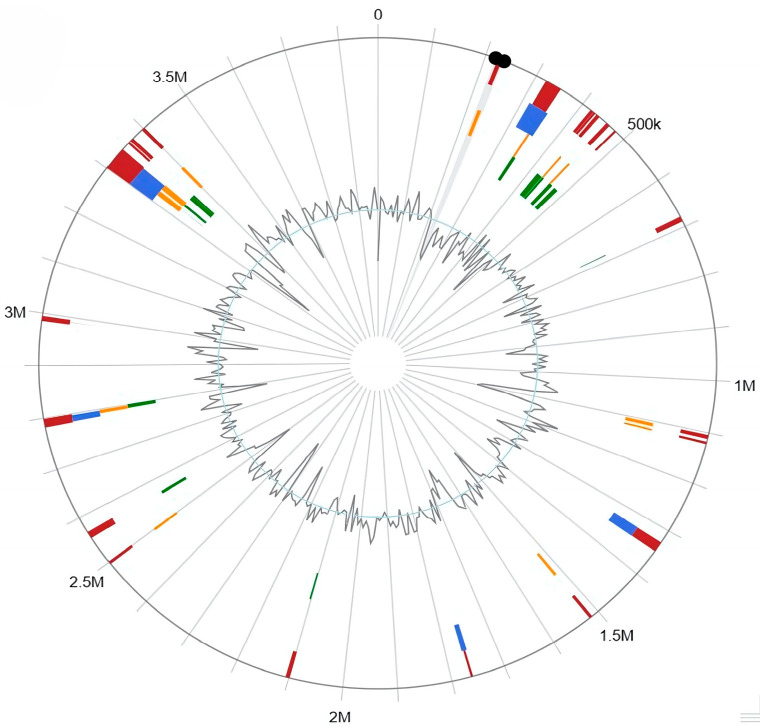
Predicted genomic islands (GIs) of *Pseudarthrobacter* sp. So.54. Red shows the prediction by the integrated approach; blue represents the results from IslandPath-DIMOB; orange displays genomic islands predicted via SIGI-HMM; green shows the results from IslandPick.

A total of 64 genomic islands (GIs) and their location in the genome of the strain were predicted by IslandViewer4 (Figure 4). The 64 GIs were composed of 392 genes, with gene distributions ranging from 222,813 to 3,343,834 bp. Among them, 301 genes expressed hypothetical nonfunctional proteins, 11 genes expressed mobile element proteins (IS3 family transposase: ISBli10, ISBli28, ISAau1, and ISAar46), and the most were related to adaptability to the Antarctic environment (Table S3).

We identified many genes associated with the metabolism of carbohydrates (*maa*, *malQ*, *sugA*, and *rbs* genes), lipids (*dagK* gene), nucleotides (*rutD*, *upp*, and *puR* genes), amino acids (*dapH* and *pat* genes), cofactors and vitamins (*thiM*, *btuD*, and *lcfB* genes), and energy (*dsbD* gene). Two GIs contained the genes *amdA* and *fmdA*, which allow bacteria to use acetamide and formamide as a carbon or nitrogen source, respectively. The *nifH* gene is also present in the genome of this strain, which plays a crucial role in N2 fixation.

Other genes (*tcrY*, *mshA*, *murQ*, *anmK*, *dasA*, *B*, *C*, and *ripB*) associated with stress defence and environmental adaptation were identified in the strain So.54. In addition, this strain contained two GIs with genes (*gsiA*, *gsiB*, *gsiC*, *gsiD*, and *ggt*) involved in these functions, as well as two GIs with genes related to heavy metal resistance, including copper, cobalt, zinc, and cadmium. Three genes within the same GI encoded the multicopper oxidases mco and MmcO, along with a copper-exporting P-type ATPase B (CopB). Other GIs contained *czcD* genes encoding cobalt-zinc-cadmium resistance proteins and *zitB* genes that codified *zinc* transporters. Additionally, six *crcB* genes related to fluorite resistance were identified in three GIs of our strain, and the *bcrA* gene encodes a transport ATP-binding protein that confers resistance to bacitracin.

### 3.4. Secondary Metabolites

**Figure 5:**
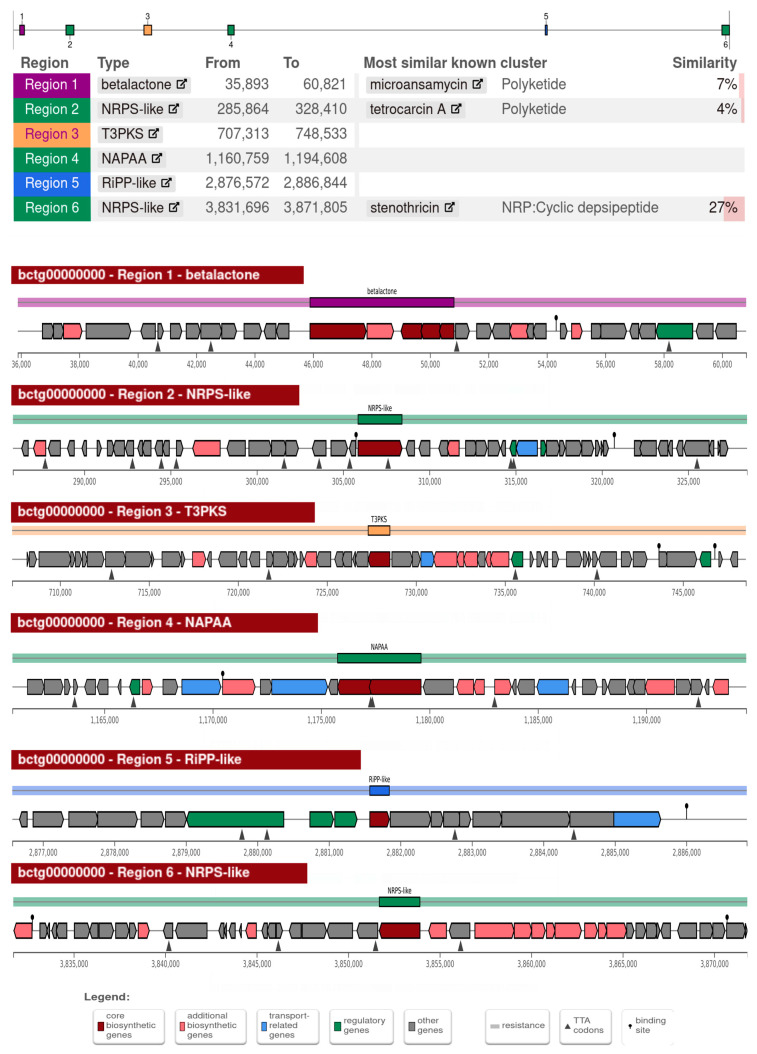
Identified secondary metabolite gene clusters in *Pseudarthrobacter* sp. So.54 using antiSMASH analysis with strictness ‘relaxed’.

To gain further insight into the potential secondary metabolites of strain So.54, secondary metabolism genes were predicted with antiSMASH tool. This analysis revealed six distinct gene clusters related to betalactone, type III polyketide synthase (T3PKS), non-alpha poly-amino acids like e-Polylysin (NAPAA), an unspecified ribosomally synthesised and post-translationally modified peptide product (RiPP-like), and two nonribosomal peptide synthetase like fragments (NRPS-like) (Figure 5). Cluster 1 (betalactone) had a high similarity of 95–100% with betalactone BGC in species of the genus *Arthrobacter* and a strain of *Pseudarthrobacter scleromae* (Figure S3). However, this cluster and the identified NRPS-like sequences exhibited low similarity (less than 50%) to known BGCs in the database. Meanwhile, T3PKS, NAPAA, and RiPP-like showed no similarity to any known BGC.

## 4. Discussion

*Pseudarthrobacter* species are found in diverse environments, including soil, water, and extreme habitats [34]. In this study, we isolated the strain So.54 from Antarctic soil, similar to *P. albicanus* NJ-25 [35] and *P. psychrotolerans* YJ56 [36], which were also isolated from Antarctic soil. According to the dDDH, ANI, and AAI results, this strain may represent a novel species within the genus *Pseudarthrobacter.* The dDDH values did not exceed the 70% threshold for species delineation. Additionally, the ANI and AAI values were below the recommended cutoff of 95–96% [37] and the proposed species boundary of 85–90% [38], respectively.

The genome of strain So.54 contained several genes related to environmental adaptation, and important complete pathways such as GABA pathway were identified. GABA plays an important role as a signaling molecule that responds to pH variation and osmotic, ionic, and cold stress [39,40]. Therefore, its production is considered a strategy to survive stressful environmental conditions. Additionally, it is known that coenzyme and vitamin metabolism play important roles in the stress adaptation/resistance mechanism. The thiamine salvage pathway and biosynthesis pathway of Pyridoxal-P, NAD, CoA, lipoic acid, and heme were completed in our strain. The potential biotechnological and contribution to the adaptation of bacteria under extreme conditions has been demonstrated in these pathways [41,42,43,44]. Essential thiamin synthetic enzymes such as ThiE (EC 2.5.1.3) are proposed as promising drug targets [45], and CoA biosynthesis has been reported as antimicrobial drug target too, acquiring biotechnological importance in our strain [46]. In addition, the PdxS enzyme (EC 4.3.3.6) present in the biosynthesis pathway of Pyridoxal-P (vitamin B6) is involved in several types of stress resistance [47].

The presence of genes involved in the non-mevalonate pathway for C5 isoprenoid biosynthesis in the strain So.54 is particularly important. In this pathway, isopentenyl diphosphate (IPP) and dimethylallyl diphosphate (DMAPP) serve as key intermediates, and their component enzymes have been genetically validated as drug targets [48]. Isoprenoids are crucial in helping bacteria adapt to various environmental stresses, including temperature fluctuations, oxidative stress, and osmotic pressure. Carotenoids, a type of isoprenoid, protect bacteria from oxidative damage by detoxifying reactive oxygen species (ROS) [49]. Additionally, due to their unique structure and antioxidant properties, carotenoid pigments are the primary agents preventing the harmful effects of UV radiation [50]. In permanently cold environments, such as Antarctica, where the temperature during the year is usually below zero and does not exceed 15 °C, carotenoids play a role in the modulation of membrane fluidity and protect bacterial cells against disruption from freezing.

The genome of strain So.54 also contains genes and multidrug efflux pumps that may confer resistance to β-lactam antibiotics and multidrug resistance, respectively. Naturally occurring antibiotic resistance genes in Antarctic surface soils mainly encode single or multidrug efflux pumps and provide inactivation of aminoglycosides, chloramphenicol, and β-lactam antibiotics—Gram-positive *Actinobacteroidota* and *Pseudomonadota* harbor 9% antibiotic resistance genes [51].

On the other hand, regarding carbohydrate metabolism, it is vital to highlight the metabolic pathway associated with the glyoxylate cycle in strain So.54. This cycle is a metabolic variation of the Krebs cycle, enabling bacteria to metabolize acetate and fatty acids while efficiently assimilating carbon sources, which is crucial for survival in diverse environments [52]. Additionally, this strain possesses metabolic pathways for biosynthesis and the degradation of polysaccharides and oligosaccharides, such as glycogen and trehalose. Bacteria with glycogen storage may have more varied lifestyles and occupy more diverse habitats and, thus, on average, will be more durable [53]. It has also been reported that trehalose is involved in bacterial adaptation to temperature fluctuation, hyperosmolarity, and desiccation resistance [9].

Given the findings from carbohydrate metabolism analysis, we further explored the carbohydrate-active enzymes (CAZymes), which are enzymes associated with the biosynthesis, binding, and catabolism of carbohydrates such as trehalose and glycogen. The enzymes identified in strain So.54 may play a crucial role in understanding the survival mechanisms of cold-adapted organisms in extreme environments and could have significant biotechnological potential. The presence of complete pathways for trehalose and glycogen metabolism in this strain is consistent with findings in *Pseudarthrobacter sulfonivorans* strain Ar51 and *Arthrobacter* sp. PAMC25564, whose enzymes are active at low temperatures and suitable for biotechnological applications [9,54].

Genes encoding osmoregulatory proteins, such as aquaporin Z and glycerol uptake facilitator protein, were also identified in strain So.54. Osmotic stress from the environment is an essential factor determining any microorganism’s ability to increase in its habitat. In a high osmotic environment, the primary response of bacteria is to accumulate various solutes at a concentration nearly equal to the osmolarity of the medium [55]. Multiple osmoregulatory transporters, such as overlapping energy coupling mechanisms, substrate specificity, and mechanosensitive channels, are known to cope with osmotic stress [56]. Aquaporins are an osmotically inducible protein released in an osmoregulatory response, which is required for growing cells. Although bacterial aquaporin proteins can theoretically contribute to osmoregulation, studies indicate that they likely function to improve freeze tolerance under rapid freezing conditions [57]. Similar to our results, the genome of the psychotrophic *Pseudarthrobacter sulfonivorans* strain Ar51 was found to have two aquaporin Z genes [54]. The presence of glycerol uptake facilitator proteins is known to occur in response to osmotic pressure. Glycerol formation and degradation should be regarded as an osmoregulatory mechanism necessary to maintain a suitable osmotic pressure within the cells [58].

Stress conditions such as cold and toxic pollutants exacerbate cellular oxidative stress, and psychrotolerants possess several molecular mechanisms to cope with these adverse conditions in the Antarctic environment [59]. As a psychrotolerant bacterium, the strain So.54 genome reflects a molecular response to environmental stress via the presence of anti-oxidative protein-coding genes. At lower temperatures, reactive oxygen species (ROS) production and associated gas solubility are more significant, and commonly linked with cellular damage. Along with ROS, lower temperature promotes activities like solubility of nutrients, diffusion reduction, stress expansion, and formation and desiccation of ice. Due to the increase in ROS, there is a high production of catalases and superoxide dismutases, considered to decrease ROS assembly [10]. The transcriptomic analysis shows the presence of superoxide reductase, 3-Cys thioredoxin peroxidase, and other proteins like anti-oxidation stress proteins and electron-transfer enzymes at lower temperatures [60]. Some of the genes responsible for ROS response under oxidative stress are *sodA* (encoding for superoxide dismutase), *tpx* (encoding for thiol peroxidases), *trxA* (encoding for thioredoxins), *osmC/ohr* (encoding for organic hydroperoxide reductase), *trxB* (encoding for thioredoxin reductase), and *katA* (encoding for catalase). These genes can be present in multiple copies in psychrophiles, contributing to their survival in cold environments and when exposed to high UV radiation [61]. In *Actinobacteria*, these enzymes have been identified, as well as proteins that regulate genes in the NO stress response and ROS production induced by heavy metals [62]. Similarly, nitric oxide (NO) is a highly reactive and toxic free radical gas that can freely diffuse into cells and attack the redox centers of proteins [63]. High concentrations of heavy metals give rise to ROS production, disrupting the redox homeostasis of cells [64].

Due to their properties, these antioxidant enzymes have become of interest in biotechnology [65,66] with a growing market demand [67]. Cold-adapted enzymes, such as those found in Antarctica, possess inherent properties that make them more effective catalysts at low temperatures. Compared to their mesophilic counterparts, these enzymes can function well under extreme conditions, offering significant market potential in biotechnological industries [68,69]. Several cold-adapted enzymes from Antarctic bacteria have been explored, including superoxide dismutase, thioredoxin, and peroxiredoxin, which have been utilized in the food, cosmetics, healthcare, and pharmaceutical industries [70,71,72,73]. The application of these enzymes has also been reported in agriculture, mainly associated with oxidative stress mitigation in plants, plant growth promotion, bioremediation, and soil protection [74,75]. All these findings suggest that our strain is of biotechnological interest.

The horizontal transfer of mobile elements in microbial communities is an essential mechanism by which bacterial genomes evolve and adapt to specific environmental stresses [76]. Hence, studying the content on the genomic islands could provide information about the evolution, adaptability, metabolic, and defense capabilities of *Pseudarthrobacter* sp. So.54. We identified several genes in this strain linked to adaptation to the Antarctic environment. Among them were the *tcrY* gene, which encodes a putative sensor histidine kinase, essential for bacterial response and adaptation to environmental changes [77], and the *mshA* gene, which encodes D-inositol-3-phosphate glycosyltransferase, playing a crucial role in the mycothiol (MSH) biosynthesis pathway. The MSH functions as a protected detoxifying cysteine reserve, detoxifying alkylating agents, reactive oxygen and nitrogen species, and antibiotics [78].

Although MSH is the major thiol found in *Actinobacteria*, this strain has two GIs with genes (*gsiA*, *gsiB*, *gsiC*, *gsiD*, and *ggt* genes) associated with the production and utilization of glutathione, protecting the cell from the action of low pH, chlorine compounds, and oxidative and osmotic stresses [79,80,81]. Cell wall, membrane, and envelope-component synthesis in bacteria are fundamental to supporting cold tolerance and protecting the cell against disruption by ice formation and osmotic pressure [82]. Some genes (*murQ*, *anmK*, *dasA*, *B*, *C*, and *ripB* genes) that respond to these functions were found in our strain.

We can also gain valuable insights into specific genes in this strain. For instance, the presence of the *nifH* gene could suggest that strain So.54 functions as a plant growth-promoting rhizobacterium, which was isolated from the rhizospheric soil of *Colobanthus quitensis*. Similar results were found in *Pseudarthrobacter oxydans* NCCP-2145, considered a halotolerant plant growth-promoting bacterium isolated from rhizospheric soil [83], and *Pseudarthrobacter chlorophenolicus* BF2P4-5, with potential as biofertilizer [84]. Other studies have also indicated that *P. oxydans*, previously characterized for their plant growth-promoting traits, could be a future biostimulant for stressed plants [85].

Genomic islands (GIs) containing genes associated with resistance to potentially toxic elements were also identified in this study. Similar to our results, several genes identified in *Arthrobacter* sp. PAMC25284 and *Pseudarthrobacter* sp. NIBRBAC000502772 were related to heavy metal resistance [1,8]. Resistance to multiple heavy metals (As^3+,^ Cd^2+^, Cr^6+^, Cu^2+^, and Ni^2+^) was also shown in isolates of *Pseudarthrobacter* and *Arthrobacter* associated with contaminated soils [86]. The acquisition of these GIs on the strain So.54 might play an important role in its survival and Antarctic rhizo-colonization. The activities of microbial communities and the enzymes involved in different metabolic processes are affected by different environmental pollutants [87]. Studies have reported the presence of contaminants in the Antarctic continent, including heavy metals, antibiotics, and pesticides transported by natural processes through the air and water. Active volcanoes also contribute a significant amount of heavy metals to the soil.

Regarding antibiotic resistance, it has been described that introducing foreign microorganisms due to tourism and scientific activities increases the acquisition of these genes by horizontal gene transfer [88]. The presence of genes related to heavy metal resistance in the strain So.54 has a relevant meaning in soil bioremediation. Some bacteria isolated from such toxic metal-contaminated/polluted sites have been found suitable for bioremediation of these sites [89]. Microbial bioremediation is much cheaper and more eco-friendly and advantageous than other conventional remediation methods [90].

In the analysis of the identified BGCs within the strain So.54, six distinct BGCs were observed. Cluster 1 showed to beta-lactone-containing protease inhibitor. Betalactone natural products are compounds isolated from bacteria known to have potent anticancer, antibiotic, and anti-obesity properties. This cluster was similar to the microansamycin biosynthesis cluster, although in a low percentage (7%). Microansamycin is a kind of ansamycin, which belongs to a family of macrolactams with remarkable bioactivities that are synthesized by type I polyketide synthase (PKS) using 3-amino-5-hydroxybenzoic acid (AHBA) as the starter unit. Most members of the family have strong antimicrobial, antifungal, anticancer, and antiviral activities [91]. Also, this cluster has a high similarity of 95–100% with betalactone BGC in species of the genus *Arthrobacter* and a strain of *Pseudarthrobacter scleromae*. Several studies in Arthrobacter sp. have reported the presence of BGCs that codify betalactone. Such is the case of *Arthrobacter wenxiniae* sp. nov., a novel plant growth-promoting rhizobacteria species [92], and *Arthrobacter* sp. GN70, which has a BGC coding for betalactone with 7% similarity to microansamycin, equal to our results [93]. Additionally, the same authors reported BGCs coding for type 3 polyketide synthase (T3PKS), non-alpha poly-amino acids such as e-Polylysin (NAPAA), and post-translationally modified peptides (RiPPs) in these species. It was similar in our case, where BGCs coding for T3PKS, NAPAA, and RiPPs were found in *Pseudarthrobacter* sp. So.54 genome. These clusters showed no similarities with known BGCs and might represent a source for studying potentially novel natural products.

Polyketides are a large class of natural products that exert pharmacologically valuable activities such as antibiotic, anticancer, immunosuppressive, and cholesterol-lowering effects [94]. Condensation reactions are catalyzed by polyketide synthases (PKSs), which are classified into three types (I, II, and III) based on their domain structures and subunit organizations. In bacteria, products of type III PKSs serve as the precursors for UV-protective pigments [95], antibiotics [96], and lipids that confer antibiotic resistance [97]. Seventeen bacterial type III PKSs have been experimentally characterized; ten of them are *Actinobacteria* [98]. In the genus *Pseudarthrobacter*, BGCs coding for T3PKS have been found. Similar to our results, it was reported in *Pseudarthrobacter sulfonivorans* strain Ar51, a bacterium with the capacity to survive extreme environmental conditions [54]. Antimicrobial properties have also been related to NAPAA; e-Polylysin is widely used as an antibacterial agent because of its broad antimicrobial spectrum [99] and RiPPs, of which 3933 biosynthetic gene clusters have been observed in *Actinobacteria* [100].

Regions 2 and 6 of the *Pseudarthrobacter* sp. So.54 genome contain BGCs coding for nonribosomal peptide synthetase (NRPS). NRPS and PKS have been studied for their antimicrobial potential. Numerous antimicrobial compounds such as beta-lactams, tetracyclines, phenazine, and aminoglycosides have already been isolated and characterized from several *Actinobacteria* and are used as drugs to control diverse human diseases. NRPS and PKS pathways are thought to be responsible for synthesizing these compounds in this group [101]. NRPS clusters in the *Pseudarthrobacter* sp. So.54 genome had similar genes with known secondary metabolites, including tetrocarcin A (4%) and stenothricin (27%). The gene cluster responsible for the production of tetrocarcin A is predominantly of polyketide origin [102]. Tetrocarcin exhibits antitumor activity and has been observed to interfere with different antiapoptotic pathways depending on cell type. In HeLa cells, tetrocarcin inhibits the mitochondrial functions of the Bcl-2 family of proteins and induces apoptosis [103]. Its bactericidal activity has also been demonstrated [104]. This action is also specific to stenothricin, an antibiotic that inhibits the cell wall synthesis of certain bacteria [105]. Several studies have shown evidence that this peptide antibiotic is produced for *Actinobacteria* such as *Arthrobacter* sp. [93,106]. Notably, the low similarity of BGCs in our strain with known clusters may represent the generation of new metabolites from a potentially novel species of *Pseudarthrobacter* sp. (So.54).

## 5. Conclusions

In this study, the whole genome of *Pseudarthrobacter* sp. So.54 was assembled, and the genomic characteristics were described by genomic analysis. The phylogenetic results confirmed that this strain belongs to the *Pseudarthrobacter* genus, phylum *Actinobacteroidota.* The low dDDH, ANI, and AAI values indicated that strain So.54 may be a new species of the genus *Pseudarthrobacter*. Our work enriches knowledge about Antarctic bacteria, describing a new strain with genomic content related to environmental adaptation that is valuable for biotechnological applications. In this sense, functional annotation and CAZyme analysis demonstrated its capacity to metabolize complex carbohydrates, including complete pathways for trehalose and glycogen, which are essential for resistance to temperature fluctuations, osmotic stress, and desiccation. Genomic island analysis identified genes associated with nitrogen fixation (*nifH*), stress adaptation (oxidative and osmotic), heavy metal resistance, and the metabolism of environmental pollutants, underscoring its biotechnological potential in bioremediation and plant growth promotion. Furthermore, the prediction of secondary metabolites revealed the presence of biosynthetic gene clusters associated with polyketides and nonribosomal peptides, which could represent sources of novel natural compounds. Together, these findings position *Pseudarthrobacter* sp. So.54 as a promising model for studying cold-adapted microorganisms with potential biotechnological applications in extreme environments.

## Figures and Tables

**Table 1:** List of enzymes in *Pseudarthrobacter* sp. So.54 associated with trehalose and glycogen metabolic pathways.

CAZyme Group	Enzyme Activity	Genes	EC Number	Number of Genes
-	ADP-glucose pyrophosphorylase	*glgC*	EC 2.7.7.27	1
GT5	Predicted glycogen synthase, ADP-glucose transglucosylase, Actinobacterial type	*glgA*	EC 2.4.1.21	2
	NDP-glucose—starch glucosyltransferase	*waxy*	EC 2.4.1.242	2
GH13 CBM48	1,4-alpha-glucan (glycogen) branching enzyme, GH-13-type	*glgB*	EC 2.4.1.18	3
-	UTP—glucose-1-phosphate uridylyltransferase	*galU*	EC 2.7.7.9	1
GT3	Glycogen synthase	*gys*	EC 2.4.1.11	1
GT35	Glycogen phosphorylase	*glgP*	EC 2.4.1.1	3
-	Phosphoglucomutase	*pgm*	EC 5.4.2.2	1
GH13 CBM48	Isoamylase/Glycogen debranching enzyme	*treX/glgX*	EC 3.2.1.68/3.2.1.	2
	Malto-oligosyltrehalose synthase	*treY*	EC 5.4.99.15	5
	Malto-oligosyltrehalose trehalohydrolase	*treZ*	EC 3.2.1.141	1

GH, glycosyl hydrolase; CBM, carbohydrate-binding modules; GT, glycosyltransferase; EC, enzyme commission number.

**Table 2:** Genes encoding stress response proteins as predicted in the genome of *Pseudarthrobacter* sp. So.54.

Gene	Start	Stop	Length (bp)	EC Number	Protein Description
*aqpZ*	1,215,433	1,214,576	858		Aquaporin Z
*glpF*	800,030	799,281	750		Glycerol uptake facilitator protein
*nsrR*	3,009,139	3,008,675	465		Nitrite-sensitive transcriptional repressor NsrR
*oxyR*	2,203,595	2,204,515	921		Hydrogen peroxide-inducible genes activator
*yaaA*	1,016,474	1,017,181	708		Peroxide stress protein YaaA
*ahpE*	1,031,338	1,030,820	519	EC:1.11.1.15	Alkyl hydroperoxide reductase E
*trxA*	468,834	469,199	366		Thioredoxin
	1,764,701	1,765,114	414		
	2,494,905	2,495,231	327		
	3,758,479	3,758,916	438		
*tpx*	2,154,742	2,155,221	480		Thioredoxin-dependent thiol peroxidase
*trxB*	2,493,849	2,494,865	1016	EC:1.8.1.9	Thioredoxin-disulfide reductase
	3,682,416	3,683,393	978		
*osmCL*	2,895,233	2,895,063	171		Organic hydroperoxide resistance transcriptional regulator
*osmCLR*	119,555	119,337	219		Organic hydroperoxide resistance protein
*sodA*	608,696	609,319	624	EC:1.15.1.1	Superoxide dismutase [Mn/Fe]
*katA*	1,782,412	1,783,911	1500	EC:1.11.1.6	Catalase
	3,440,614	3,441,678	1065		

## Data Availability

The original contributions presented in the study are included in the article/Supplementary Material, further inquiries can be directed to the corresponding authors.

## Supplementary Materials

The following supporting information can be downloaded at: https://www.mdpi.com/article/10.3390/biom15040534/s1, Figure S1: Pairwise comparisons of the average nucleotide identity (A) and average amino acid identity (B) between genome sequencing of strain So.54 (highlighted within a blue box) and representative species of *Pseudarthrobacter* and *Arthrobacter* genus; Figure S2: Predicted pathways for trehalose and glycogen metabolism in *Pseudarthrobacter* sp. So.54 as a cold adaptation response. KEGG annotation database with the Anvi’o platform- predicted enzyme pathways (green squares) and dbCAN3-predicted CAZyme family (orange squares). Trehalose biosynthesis pathway (black line), glycogen biosynthesis pathway (red line) and glycogen degradation pathway (blue line); Figure S3: Similarity plots of betalactone BGC of *Pseudarthrobacter* sp. So.54 (highlighted) with species of the *Arthrobacter* and *Pseudarthrobacter* genus. The colors match the shared genes; Table S1: Comparison of strain So.54 with *Pseudarthrobacter* sp. and *Arthrobacter* sp. concerning digital hybridization ADN-ADN (dDDH) values and G+C content difference. dDDH formula d4: references values recommended by TYGS; Table S2: Genes potentially involved in metabolic pathways associated with environmental adaptation in the strain So.54; Table S3: Functional protein-producing genes identified in genomic islands of *Pseudarthrobacter* sp. So.54 by IslandViewer4.
