# Supplementary material for: Genome Mining of Pseudarthrobacter sp. So.54, a Rhizospheric Bacteria from Colobanthus quitensis Antarctic Plant
Source: Biomolecules. 2025 Apr 5;15(4):534. doi: 10.3390/biom15040534 (PMC12025171; doi:10.3390/biom15040534)
Supplement: Supplementary file 1 [file biomolecules-15-00534-s001.zip › biomolecules-3549907-supplementary.pdf]

Supplementary material

Genome mining of *Pseudarthrobacter* sp. So.54, a rhizospheric bacteria from *Colobanthus quitensis* Antarctic plant

Dayaimi González[1,2], Pablo Bruna[1,3], María J. Contreras[4], Karla Leal[4], Catherine V. Urrutia[1,2], Kattia Núñez-Montero[5,*], Leticia Barrientos[5,*]

[1] Programa de Doctorado en Ciencias mención Biología Celular y Molecular Aplicada, Universidad de La Frontera, Temuco, Chile; c.urrutia08@ufromail.cl (C.V.U.)

[2] Centro de Excelencia en Medicina Traslacional (CEMT), Universidad de La Frontera, Avenida Alemania 0458, Temuco, Chile

[3] Núcleo Científico y Tecnológico en Biorecursos (BIOREN), Universidad de La Frontera, Avenida Francisco Salazar 01145, Temuco, Chile

[4] Facultad de Ciencias de la Salud, Instituto de Ciencias Aplicadas, Universidad Autónoma de Chile, Avenida Alemania 1090, Temuco, Chile

[5] Facultad de Ingeniería, Instituto de Ciencias Aplicadas, Universidad Autónoma de Chile, Avenida Alemania 1090, Temuco, Chile

* Correspondence: kattia.nunez@uautonoma.cl (K.N.-M.); leticia.barrientos@uautonoma.cl (L.B.); Tel.: +56-9-90101034 (L.B.)

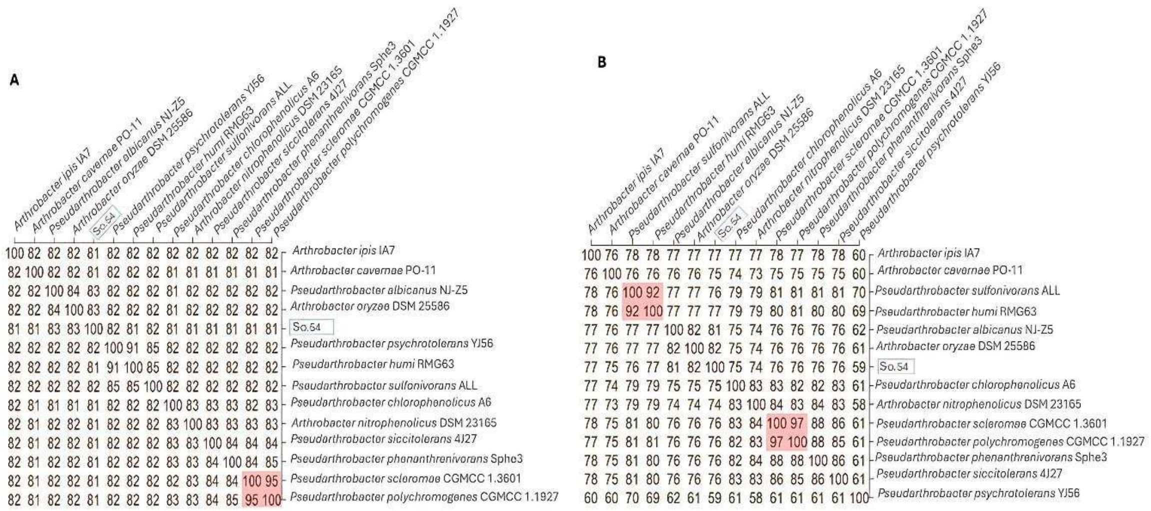

Figure S1. Pairwise comparisons of the average nucleotide identity **(A)** and average amino acid identity **(B)** between genome sequencing of strain So.54 (highlighted within a blue box) and representative species of *Pseudarthrobacter* and *Arthrobacter* genus.

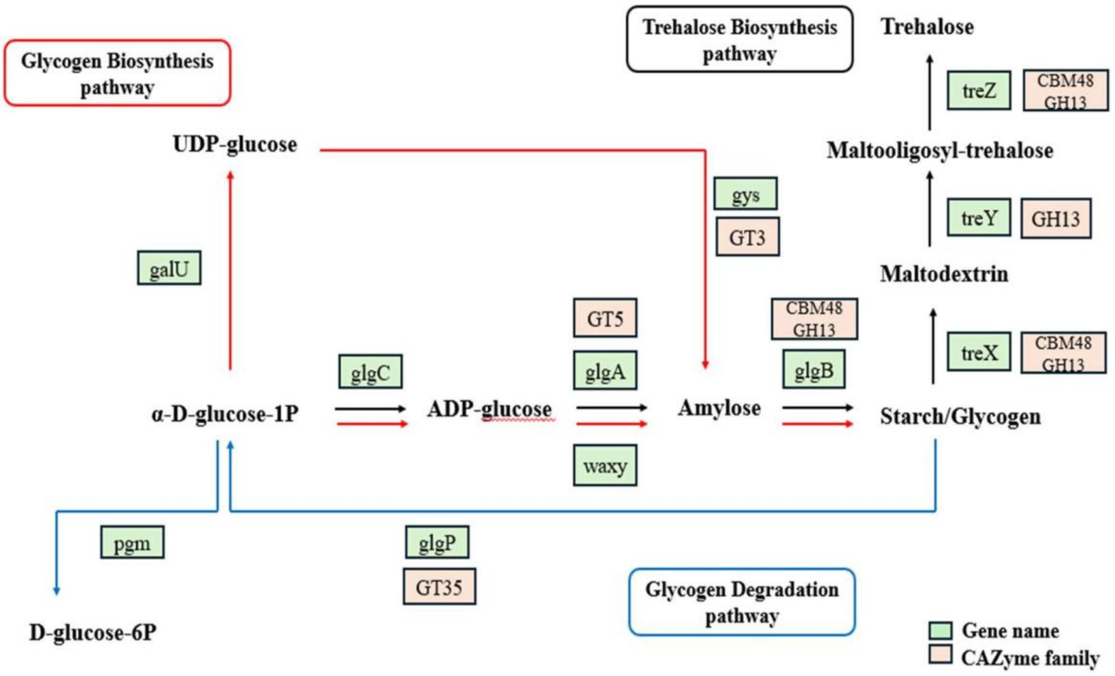

Figure S2. Predicted pathways for trehalose and glycogen metabolism in *Pseudarthrobacter* sp. So.54 as a cold adaptation response. KEGG annotation database with the Anvi'o platform- predicted enzyme pathways (green squares) and dbCAN3-predicted CAZyme family (orange squares). Trehalose biosynthesis pathway (black line), glycogen biosynthesis pathway (red line) and glycogen degradation pathway (blue line).

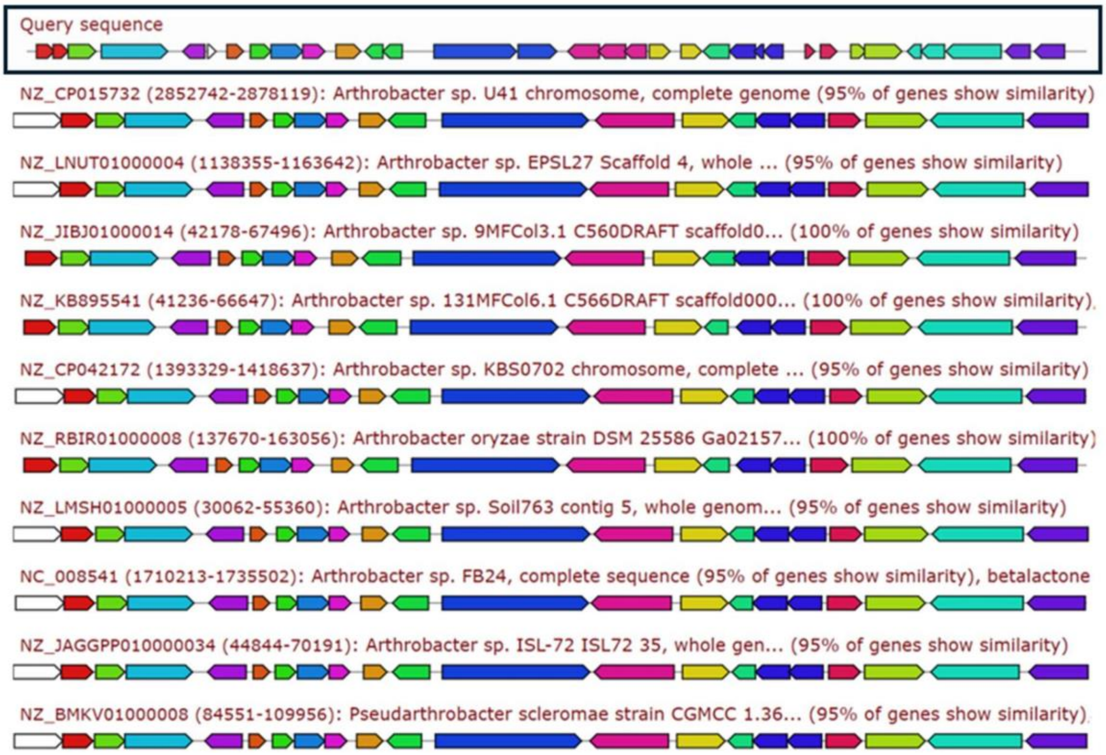

Figure S3. Similarity plots of betalactone BGC of *Pseudarthrobacter* sp. So.54 (highlighted) with species of the *Arthrobacter* and *Pseudarthrobacter* genus. The colors match the shared genes.

Table S1. Comparison of strain So.54 with Pseudarthrobacter sp. and Arthrobacter sp. concerning digital hybridization ADN-ADN (dDDH) values and G+C content difference. dDDH formula d4: references values recommended by TYGS.

Query strain	Subject strain	dDDH (d4,%)	C.I. (d4,%)
'So.54'	*Pseudarthrobacter albicanus* NJ-Z5	25.3	[22.9-27.7]
'So.54'	*Arthrobacter oryzae* DSM 25586	24.6	[22.3-27.1]
'So.54'	*Pseudarthrobacter psychrotolerans* YJ56	22.6	[20.3-25.0]
'So.54'	*Arthrobacter ipis* IA7	22.5	[20.2-25.0]
'So.54'	*Pseudarthrobacter humi* RMG13	22.4	[20.1-24.8]
'So.54'	*Pseudarthrobacter sulfonivorans* ALL	22.4	[20.1-24.9]
'So.54'	*Pseudarthrobacter polychromogenes* CGMCC 1.1927	22.0	[19.8-24.5]
'So.54'	*Arthrobacter nitrophenolicus* SJConT	22.0	[19.7-24.4]
'So.54'	*Arthrobacter cavernae* PO-11	22.0	[19.8-24.5]
'So.54'	*Pseudarthrobacter scleromae* CGMCC 1.3601	21.9	[19.7-24.4]
'So.54'	*Pseudarthrobacter phenanthrenivorans* Sphe3	21.9	[19.7-24.4]
'So.54'	*Pseudarthrobacter siccitolerans* 4J27	21.8	[19.6-24.3]
'So.54'	*Arthrobacter bangladeshi* MAHUQ-56	21.8	[19.5-24.2]
'So.54'	*Pseudarthrobacter chlorophenolicus* A6	21.7	[19.4-24.1]

Table S2. Genes potentially involved in metabolic pathways associated with environmental adaptation in the strain So.54.

KEGG Module	Metabolic pathway	Gene	Enzyme/ Multidrug efflux pumps	EC number
M00165	Reductive pentose phosphate cycle	*gapA*<br>*tkt*<br>*pgk*<br>*fbaA*<br>*rpe*<br>*tpiA*<br>*rpiB*<br>*glpX*	glyceraldehyde 3-phosphate dehydrogenase<br>transketolase<br>phosphoglycerate kinase<br>fructose-bisphosphate aldolase<br>triosephosphate isomerase<br>ribose 5-phosphate isomerase B<br>fructose-1,6-bisphosphatase II	EC:1.2.1.12<br>EC:2.2.1.1<br>EC:2.7.2.3<br>EC:4.1.2.13<br>EC:5.3.1.1<br>EC:5.3.1.6<br>EC:3.1.3.11
M00579	Phosphate acetyltransferase-acetate kinase	*ackA*	acetate kinase	EC:2.7.2.1

	pathway, acetyl-CoA => acetate			
M00345	Formaldehyde assimilation, ribulose monophosphate pathway	*fbaA*<br>*pfkB*<br>*pfk*<br>*pfp*	fructose-bisphosphate aldolase, class II<br>6-phosphofructokinase 2<br>ATP-dependent<br>phosphofructokinase / diphosphate-dependent phosphofructokinase	EC:4.1.2.13<br>EC:2.7.1.11<br>EC:2.7.1.11<br>EC:2.7.1.90
M00176	Assimilatory sulfate reduction, sulfate => H_2S	*cysH*<br>*sir*<br>*cysN*<br>*cysD*	phosphoadenosine phosphosulfate reductase<br>(thioredoxin)<br>sulfite reductase (ferredoxin)<br>sulfate adenylyltransferase subunit 1<br>sulfate adenylyltransferase subunit 2	EC:1.8.4.8<br>EC:1.8.7.1<br>EC:2.7.7.4
M00155	Cytochrome c oxidase	*coxA, ctaD*<br>*coxB, ctaC*<br>*coxC, ctaE*<br>*ctaF*	cytochrome c oxidase subunit I<br>cytochrome c oxidase subunit II<br>cytochrome c oxidase subunit III<br>cytochrome c oxidase subunit IV	EC:7.1.1.9
M00157	F-type ATPase	*atpB*<br>*atpF*<br>*atpE*<br>*atpA*<br>*atpD*<br>*atpH*<br>*atpC*<br>*atpG*	F-type H+-transporting ATPase subunit a<br>F-type H+-transporting ATPase subunit b<br>F-type H+-transporting ATPase subunit c<br>F-type H+/Na+-transporting ATPase subunit alpha<br>F-type H+/Na+-transporting ATPase subunit beta<br>F-type H+-transporting ATPase subunit delta<br>F-type H+-transporting ATPase subunit epsilon<br>F-type H+-transporting ATPase subunit gamma	-<br>EC: 7.2.2.1
M00086	beta-Oxidation, acyl-CoA synthesis	*fadD*	long-chain acyl-CoA synthetase	EC:6.2.1.3
M00027	GABA (gamma-Aminobutyrate) shunt	*gabD*<br>*gad*<br>*gabT*	succinate-semialdehyde dehydrogenase / glutarate-semialdehyde dehydrogenase<br>glutamate decarboxylase<br>4-aminobutyrate aminotransferase / (S)-3-amino-2-	EC:2.5.1.32<br>EC:1.2.1.16<br>EC:1.2.1.79<br>EC:1.2.1.20<br>EC:4.1.1.15<br>EC:2.6.1.19<br>EC:2.6.1.22<br>EC:2.6.1.48

			methylpropionate transaminase / 5-aminovalerate transaminase	
M00899	Thiamine salvage pathway	*thiE*	thiamine-phosphate pyrophosphorylase	EC:2.5.1.3
		thiM	hydroxyethylthiazole kinase	EC:2.7.1.50
		thiD	hydroxymethylpyrimidine/phosp homethylpyrimidine kinase	EC:2.7.1.49 EC:2.7.4.7
M00916	Pyridoxal-P biosynthesis, R5P + glyceraldehyde-3P + glutamine => pyridoxal-P	*pdxS*	pyridoxal 5'-phosphate synthase pdxS subunit	EC:4.3.3.6
		pdxT	pyridoxal 5'-phosphate synthase pdxT subunit	EC:4.3.3.6
M00115	NAD biosynthesis, aspartate => quinolinate => NAD	*nadB*	L-aspartate oxidase	EC:1.4.3.16
			nicotinate-nucleotide	
		nadC	pyrophosphorylase	EC:2.4.2.19
		nadD	(carboxylating)	EC:2.7.7.18
			nicotinate-nucleotide	
		nadE	adenylyltransferase	EC:6.3.1.5
		nadA	NAD+ synthase quinolinate synthase	EC:2.5.1.72
M00120	Coenzyme A biosynthesis, pantothenate => CoA	*coaE*	dephospho-CoA kinase	EC:2.7.1.24
		coaA	type I pantothenate kinase	EC:2.7.1.33
		coaD	pantetheine-phosphate adenylyltransferase	EC:2.7.7.3
		coaBC	phosphopantothenoylcysteine	EC:4.1.1.36
			decarboxylase / phosphopantothenate---cysteine ligase	EC:6.3.2.5
M00881	Lipoic acid biosynthesis	*lipA*	lipoyl synthase	EC:2.8.1.8
		lipB	lipoyl(octanoyl) transferase	EC:2.3.1.181
M00926	Heme biosynthesis, bacteria, glutamyl-tRNA => coproporphyrin III => heme	*hemY*	protoporphyrinogen/coproporph yrinogen III oxidase	EC:1.3.3.4 EC:1.3.3.15
		hemQ	hydrogen peroxide-dependent heme synthase	EC:1.3.98.5
		hemE	uroporphyrinogen decarboxylase	EC:4.1.1.37
		hemB	porphobilinogen synthase	EC:4.2.1.24
		hemD	uroporphyrinogen-III synthase	EC:4.2.1.75
		hemC	hydroxymethylbilane synthase	EC:2.5.1.61
		hemH	protoporphyrin/coproporphyrin ferrochelatase	EC:4.98.1.1 EC:4.99.1.9
		hemL	glutamate-1-semialdehyde 2,1-aminomutase	EC:5.4.3.8
		hemA	glutamyl-tRNA reductase	EC:1.2.1.70
M00140	C1-unit interconversion	*glyA*	glycine hydroxymethyltransferase	EC:2.1.2.1
		folD	methylenetetrahydrofolate dehydrogenase (NADP+) / methenyltetrahydrofolate cyclohydrolase	EC:1.5.1.5 EC:3.5.4.9
		fhs	formate--tetrahydrofolate ligase	EC:6.3.4.3

M00096	C5 isoprenoid biosynthesis, non-mevalonate pathway	*dxr*	1-deoxy-D-xylulose-5-phosphate reductoisomerase	EC:1.1.1.267
		ispE	4-diphosphocytidyl-2-C-methyl-D-erythritol kinase	EC:2.7.1.148
		dxs	1-deoxy-D-xylulose-5-phosphate synthase	EC:2.2.1.7
		ispF	2-C-methyl-D-erythritol 2,4-cyclodiphosphate synthase	EC:4.6.1.12
		ispG	(E)-4-hydroxy-3-methylbut-2-enyl-diphosphate synthase	EC:1.17.7.1 EC:1.17.7.3
		ispH	4-hydroxy-3-methylbut-2-en-1-yl diphosphate reductase	EC:1.17.7.4
M00538	Toluene degradation, toluene => benzoate	*xylC*	benzaldehyde dehydrogenase (NAD)	EC:1.2.1.28
M00537	Xylene degradation, xylene => methylbenzoate			
M00568	Catechol ortho-cleavage, catechol => 3-oxoadipate	*pcaL*	3-oxoadipate enol-lactonase / 4-carboxymuconolactone decarboxylase	EC:3.1.1.24 E:4.1.1.44
M00878	Phenylacetate degradation, phenylaxetate => acetyl-CoA/succinyl-CoA	*paaH*	3-hydroxybutyryl-CoA dehydrogenase	EC:1.1.1.157
		paaF	enoyl-CoA hydratase	EC:4.2.1.17
		paaK	phenylacetate-CoA ligase	EC:6.2.1.30
		paaA	ring-1,2-phenylacetyl-CoA epoxidase subunit PaaA	EC:1.14.13.149
		paaB	ring-1,2-phenylacetyl-CoA epoxidase subunit PaaB	
		paaC	ring-1,2-phenylacetyl-CoA epoxidase subunit PaaC	
		paaD	ring-1,2-phenylacetyl-CoA epoxidase subunit PaaD	
		paaE	ring-1,2-phenylacetyl-CoA epoxidase subunit PaaE	EC:3.3.2.12
		paaZ	oxepin-CoA hydrolase / 3-oxo-5,6-dehydrosuberyl-CoA semialdehyde dehydrogenase	EC:1.2.1.91 EC:5.3.3.18
		paaG	2-(1,2-epoxy-1,2-dihydrophenyl)acetyl-CoA isomerase	
M00627	beta-Lactam resistance, Bla system	*penP*	beta-lactamase class A	EC:3.5.2.6
M00639	Multidrug resistance, efflux pump MexCD-OprJ	*nfxB*	TetR/AcrR family transcriptional regulator, mexCD-oprJ operon repressor	-

M00769	Multidrug resistance, efflux pump MexPQ-OpmE	*cueR*	MerR family transcriptional regulator, copper efflux regulator	-
M00714	Multidrug resistance, efflux pump QacA	*qacA*	MFS transporter, DHA2 family, multidrug resistance protein	-
M00001	Glycolysis (Embden-Meyerhof pathway), glucose => pyruvate	*gapA*	glyceraldehyde 3-phosphate dehydrogenase (phosphorylating)	EC:1.2.1.12
		pyk	pyruvate kinase	EC:2.7.1.40
		ppgK	polyphosphate glucokinase	EC:2.7.1.63
		pgk	phosphoglycerate kinase	EC:2.7.2.3
		fbaA	fructose-bisphosphate aldolase, class II	EC:4.1.2.13
		eno	enolase	EC:4.2.1.11
		tpiA	triosephosphate isomerase	EC:5.3.1.1
		pgi	glucose-6-phosphate isomerase	EC:5.3.1.9
		gpmA	2,3-bisphosphoglycerate-dependent phosphoglycerate	EC:5.4.2.11
		pfkB	mutase	EC:2.7.1.11
		pfk, pfp	6-phosphofructokinase 2	EC:2.7.1.11
			ATP-dependent phosphofructokinase / diphosphate-dependent phosphofructokinase	EC:2.7.1.90
M00003	Gluconeogenesis, oxaloacetate => fructose-6P	*gapA*	glyceraldehyde 3-phosphate dehydrogenase (phosphorylating)	EC:1.2.1.12
		pgk	phosphoglycerate kinase	EC:2.7.2.3
		pckA	phosphoenolpyruvate	EC:4.1.1.32
		fbaA	carboxykinase	EC:4.1.2.13
			fructose-bisphosphate aldolase,	
		eno	class II	EC:4.2.1.11
		tpiA	enolase	EC:5.3.1.1
		gpmA	triosephosphate isomerase	EC:5.4.2.11
			2,3-bisphosphoglycerate-	
		glpX	dependent phosphoglycerate	EC:3.1.3.11
			mutase	
			fructose-1,6-bisphosphatase II	
M00009	Citrate cycle (TCA cycle, Krebs cycle)	*icd*	isocitrate dehydrogenase	EC:1.1.1.42
		mqo	malate dehydrogenase (quinone)	EC:1.1.5.4
		sdhB	succinate dehydrogenase iron-sulfur subunit	EC:1.3.5.1
		sdhC	succinate dehydrogenase cytochrome b subunit	
		sdhD	succinate dehydrogenase membrane anchor subunit	
		lpd	ihydrolipoyl dehydrogenase	EC:1.8.1.4
			multifunctional 2-oxoglutarate	
		kgd	metabolism enzyme	EC:2.2.1.5<br>EC:4.1.1.71

				EC:1.2.4.2
			2-methylcitrate synthase	EC:2.3.1.61
		prpC	fumarate hydratase, class II	EC:2.3.3.5
		fumC	succinyl-CoA synthetase alpha	EC:4.2.1.2
		sucD	subunit	EC:6.2.1.5
			succinyl-CoA synthetase beta	
		sucC	subunit	EC:6.2.1.5
			aconitate hydratase A / 2-	
		acnA	methylisocitrate dehydratase	EC:4.2.1.3
				EC:4.2.1.99
M00006	Pentose phosphate	*gnd*	6-phosphogluconate	EC:1.1.1.44
	pathway, oxidative	*zwf*	dehydrogenase	EC:1.1.1.49
	phase, glucose 6P		glucose-6-phosphate 1-	
	=> ribulose 5P	*pgl*	dehydrogenase	EC:3.1.1.31
			6-phosphogluconolactonase	
M00007	Pentose phosphate	*tkt*	transketolase	EC:2.2.1.1
	pathway, non-	*tal*	transaldolase	EC:2.2.1.2
	oxidative phase,	*rpe*	ribulose-phosphate 3-epimerase	EC:5.1.3.1
	fructose 6P =>	*rpiB*	ribose 5-phosphate isomerase B	EC:5.3.1.6
	ribose 5P			
M00632	Galactose	*galK*	galactokinase	EC:2.7.1.6
	degradation, Leloir	*galT*	UDPglucose--hexose-1-phosphate	EC:2.7.7.12
	pathway, galactose		uridylyltransferase	
	=> alpha-D-glucose-	*galE*	UDP-glucose 4-epimerase	EC:5.1.3.2
	1P	*galM*	aldose 1-epimerase	EC:5.1.3.3
M00308	Semi-	*gapA*	glyceraldehyde 3-phosphate	EC:1.2.1.12
	phosphorylative		dehydrogenase (phosphorylating)	
	Entner-Doudoroff	*kdgK*	2-dehydro-3-deoxygluconokinase	EC:2.7.1.45
	pathway, gluconate	*pgk*	phosphoglycerate kinase	EC:2.7.2.3
	=> glycerate-3P	*eda*	2-dehydro-3-	EC:4.1.2.14
			deoxyphosphogluconate aldolase	EC:4.1.3.42
			/ (4S)-4-hydroxy-2-oxoglutarate	
			aldolase	
M00012	Glyoxylate cycle	*aceA*	isocitrate/methylisocitrate lyase	EC:4.1.3.1
				EC.4.1.3.30
		aceB	malate synthase	EC:2.3.3.9
		prpC	2-methylcitrate synthase	EC:2.3.3.5
		acnA	aconitate hydratase A / 2-	EC:4.2.1.3
			methylisocitrate dehydratase	EC:4.2.1.99

Table S3. Functional protein-producing genes identified in genomic islands of *Pseudarthrobacter* sp. So.54 by IslandViewer4.

Island start	Island end	Length	Gene ID	Locus	Gene start	Gene end	Product
319951	350784	30833	*mco_1*	HCKKLEIA_00388	324743	326212	Multicopper oxidase mco
319951	350784	30833	*lgt_2*	HCKKLEIA_00394	330256	331149	Phosphatidylglycerol--prolipoprotein diacylglyceryl transferase
319951	350784	30833	*mmcO*	HCKKLEIA_00395	331162	332181	Multicopper oxidase MmcO
319951	350784	30833	*dsbD*	HCKKLEIA_00397	332716	333465	Thiol:disulfide interchange protein DsbD
319951	350784	30833	*copB_1*	HCKKLEIA_00401	335758	337887	Copper-exporting P-type ATPase B
319951	350784	30833	*crcB_1*	HCKKLEIA_00413	347838	348383	Putative fluoride ion transporter CrcB
319951	350784	30833	*crcB_2*	HCKKLEIA_00414	348380	348751	Putative fluoride ion transporter CrcB
319951	350784	30833	*amdA_1*	HCKKLEIA_00417	350035	350784	Acetamidase
319951	350784	30833	*fmdA*	HCKKLEIA_00418	350745	351290	Formamidase
344515	349684	5169	*crcB_1*	HCKKLEIA_00413	347838	348383	Putative fluoride ion transporter CrcB
344515	349684	5169	*crcB_2*	HCKKLEIA_00414	348380	348751	Putative fluoride ion transporter CrcB
345788	351393	5605	*crcB_1*	HCKKLEIA_00413	347838	348383	Putative fluoride ion transporter CrcB
345788	351393	5605	*crcB_2*	HCKKLEIA_00414	348380	348751	Putative fluoride ion transporter CrcB
345788	351393	5605	*amdA_1*	HCKKLEIA_00417	350035	350784	Acetamidase
345788	351393	5605	*fmdA*	HCKKLEIA_00418	350745	351290	Formamidase
432214	436542	4328		HCKKLEIA_00516	433155	433781	IS3 family transposase ISBli10
432525	439293	6768		HCKKLEIA_00516	433155	433781	IS3 family transposase ISBli10

432525	439293	6768	*maa*	HCKKLEIA_00521	436646	437239	Maltose O-acetyltransferase
432525	439293	6768		HCKKLEIA_00524	439234	440421	putative membrane protein
455997	460836	4839	*thiM*	HCKKLEIA_00546	455997	456686	Hydroxyethylthiazole kinase
455997	460836	4839	*thiE_1*	HCKKLEIA_00547	456683	457390	Thiamine-phosphate synthase
455997	460836	4839	*bluF*	HCKKLEIA_00550	458998	459753	Blue light- and temperature-regulated antirepressor BluF
458703	463779	5076	*bluF*	HCKKLEIA_00550	458998	459753	Blue light- and temperature-regulated antirepressor BluF
458703	463779	5076		HCKKLEIA_00552	461154	461780	N-acetyldiaminopimelate deacetylase
476173	480496	4323	*wapA*	HCKKLEIA_00577	479003	480025	tRNA(Glu)-specific nuclease WapA
679148	689627	10479	*malQ_2*	HCKKLEIA_00821	678913	679305	4-alpha-glucanotransferase
679148	689627	10479	*rutD*	HCKKLEIA_00824	680391	682922	Putative aminoacrylate hydrolase RutD
679148	689627	10479	*nanR_1*	HCKKLEIA_00825	683012	683782	HTH-type transcriptional repressor NanR
1117097	1121694	4597	*btuD_3*	HCKKLEIA_01330	1118190	1119089	Vitamin B12 import ATP-binding protein BtuD
1117097	1121694	4597	*mshA_2*	HCKKLEIA_01332	1119954	1121051	D-inositol-3-phosphate glycosyltransferase
1513810	1520142	6332	*dapH_2*	HCKKLEIA_01799	1515723	1516367	2,3,4,5-tetrahydropyridine-2,6-dicarboxylate N-acetyltransferase
1760242	1764673	4431		HCKKLEIA_02075	1760242	1761144	IS3 family transposase ISBli28
1760242	1764673	4431	*lcfB_2*	HCKKLEIA_02079	1762783	1763778	Long-chain-fatty-acid--CoA ligase
2098481	2106401	7920	*ccpA_1*	HCKKLEIA_02486	2099268	2099924	Catabolite control protein A
2098481	2106401	7920	*rbsK*	HCKKLEIA_02488	2100316	2101059	Ribokinase
2098481	2106401	7920	*rbsD*	HCKKLEIA_02490	2101460	2101753	D-ribose pyranase

2098481	2106401	7920	*rbsA_1*	HCKKLEIA_02491	2101755	2103245	Ribose import ATP-binding protein RbsA
2098481	2106401	7920	*rbsC*	HCKKLEIA_02492	2103242	2104204	Ribose import permease protein RbsC
2098481	2106401	7920	*rbsB*	HCKKLEIA_02493	2104690	2105235	Ribose import binding protein RbsB
2098481	2106401	7920	*deoC_1*	HCKKLEIA_02494	2105271	2105939	Deoxyribose-phosphate aldolase
2496217	2501897	5680	*noc*	HCKKLEIA_02966	2499539	2500762	Nucleoid occlusion protein
2496217	2501897	5680	*nifH*	HCKKLEIA_02967	2500830	2501897	Nitrogenase iron protein
2555808	2569062	13254	*nagB_2*	HCKKLEIA_03032	2555241	2556026	Glucosamine-6-phosphate deaminase
2555808	2569062	13254	*ybbH_1*	HCKKLEIA_03033	2556023	2556910	putative HTH-type transcriptional regulator YbbH
2555808	2569062	13254	*murQ*	HCKKLEIA_03034	2556900	2557874	N-acetylmuramic acid 6-phosphate etherase
2555808	2569062	13254	*anmK*	HCKKLEIA_03036	2558784	2560055	Anhydro-N-acetylmuramic acid kinase
2555808	2569062	13254	*nagZ*	HCKKLEIA_03037	2560164	2561564	Beta-hexosaminidase
2555808	2569062	13254	*dasC*	HCKKLEIA_03038	2561561	2562136	Diacetylchitobiose uptake system permease protein DasC
2555808	2569062	13254	*dasB*	HCKKLEIA_03040	2562387	2563271	Diacetylchitobiose uptake system permease protein DasB
2555808	2569062	13254	*dasA_1*	HCKKLEIA_03041	2563363	2564658	Diacetylchitobiose binding protein DasA
2555808	2569062	13254	*agaA*	HCKKLEIA_03042	2564907	2565248	N-acetylgalactosamine-6-phosphate deacetylase
2555808	2569062	13254	*nagA*	HCKKLEIA_03043	2565226	2565501	N-acetylglucosamine-6-phosphate deacetylase

2555808	2569062	13254	*ulaR*	HCKKLEIA_03045	2565923	2566894	HTH-type transcriptional regulator UlaR
2781860	2798200	16340	*bcrA*	HCKKLEIA_03311	2781860	2782414	Bacitracin transport ATP-binding protein BcrA
2781860	2798200	16340	*ripB*	HCKKLEIA_03314	2783532	2783879	Peptidoglycan endopeptidase RipB
2781860	2798200	16340	*czcD_2*	HCKKLEIA_03316	2785273	2785932	Metal cation efflux system protein CzcD
2781860	2798200	16340	*zitB_2*	HCKKLEIA_03317	2785899	2786225	Zinc transporter ZitB
2781860	2798200	16340	*paiB*	HCKKLEIA_03322	2788316	2788699	Protease synthase and sporulation protein PAI 2
2781860	2798200	16340		HCKKLEIA_03331	2792240	2792563	IS3 family transposase ISAau1
2781860	2798200	16340		HCKKLEIA_03332	2793068	2793346	IS3 family transposase ISAau1
2790115	2798200	8085		HCKKLEIA_03331	2792240	2792563	IS3 family transposase ISAau1
2790115	2798200	8085		HCKKLEIA_03332	2793068	2793346	IS3 family transposase ISAau1
2984923	2994809	9886		HCKKLEIA_03575	2984923	2985342	IS3 family transposase ISAar46
2984923	2994809	9886		HCKKLEIA_03576	2985339	2985782	IS3 family transposase ISBli10
2984923	2994809	9886	*relJ*	HCKKLEIA_03580	2986542	2986790	Antitoxin RelJ
2984923	2994809	9886	*tadA_3*	HCKKLEIA_03588	2990364	2990567	tRNA-specific adenosine deaminase
2984923	2994809	9886	*tadA_4*	HCKKLEIA_03589	2990585	2990866	tRNA-specific adenosine deaminase
2984923	2994809	9886	*upp*	HCKKLEIA_03591	2991807	2992442	Uracil phosphoribosyltransferase
2984923	2994809	9886	*tcrY_1*	HCKKLEIA_03594	2994069	2994809	putative sensor histidine kinase TcrY
3301003	3343834	42831	*pat_2*	HCKKLEIA_03968	3303776	3304552	Putative phenylalanine aminotransferase
3301003	3343834	42831	*pat_3*	HCKKLEIA_03969	3304549	3304878	Putative phenylalanine aminotransferase

3301003	3343834	42831	*gabP_5*	HCKKLEIA_03970	3304920	3306329	GABA permease
3301003	3343834	42831	*malL_5*	HCKKLEIA_03977	3310449	3312146	Oligo-1,6-glucosidase
3301003	3343834	42831	*sugA_3*	HCKKLEIA_03979	3312994	3313941	Trehalose transport system permease protein SugA
3301003	3343834	42831		HCKKLEIA_03980	3314136	3314699	putative ABC transporter-binding protein
3301003	3343834	42831	*ccpA_4*	HCKKLEIA_03982	3315570	3316127	Catabolite control protein A
3301003	3343834	42831	*purR_2*	HCKKLEIA_03983	3316210	3316587	HTH-type transcriptional repressor PurR
3301003	3343834	42831	*dagK_5*	HCKKLEIA_03985	3317123	3317431	Diacylglycerol kinase
3301003	3343834	42831		HCKKLEIA_04000	3328634	3329053	IS3 family transposase ISAar46
3301003	3343834	42831	*naeIR*	HCKKLEIA_04011	3342908	3343834	Type-2 restriction enzyme NaeI
3317123	3328408	11285	*dagK_5*	HCKKLEIA_03985	3317123	3317431	Diacylglycerol kinase
3320404	3329869	9465		HCKKLEIA_04000	3328634	3329053	IS3 family transposase ISAar46
3332084	3344601	12517	*naeIR*	HCKKLEIA_04011	3342908	3343834	Type-2 restriction enzyme NaeI
3333021	3345938	12917	*naeIR*	HCKKLEIA_04011	3342908	3343834	Type-2 restriction enzyme NaeI
3360521	3365090	4569		HCKKLEIA_04030	3359669	3360640	Putative hydrolase/MSMEI_3903
3360521	3365090	4569	*gsiB*	HCKKLEIA_04032	3361619	3363205	Glutathione-binding protein GsiB
3360521	3365090	4569	*gsiC_1*	HCKKLEIA_04034	3363627	3364166	Glutathione transport system permease protein GsiC
3360521	3365090	4569	*gsiD_2*	HCKKLEIA_04035	3364167	3365060	Glutathione transport system permease protein GsiD
3366723	3373955	7232	*ggt*	HCKKLEIA_04036	3365660	3366895	Glutathione hydrolase proenzyme

3366723	3373955	7232	*gsiA_3*	HCKKLEIA_04038	3366943	3368322	Glutathione import ATP-binding protein GsiA
